# Sequential Change Detection with Local Differential Privacy

**DOI:** 10.3390/e28040402

**Published:** 2026-04-02

**Authors:** Lixing Zhang, Xuran Liu, Ruizhi Zhang, Liyan Xie

**Affiliations:** 1 Department of Industrial and Systems Engineering, University of Minnesota, Minneapolis, MN 55455, USA; zhan9503@umn.edu; 2 Department of Statistics, University of Georgia, Athens, GA 30602, USA; xuran.liu@uga.edu (X.L.); ruizhi.zhang@uga.edu (R.Z.)

**Keywords:** sequential change detection, local differential privacy, cumulative sum test, average run length, detection delay

## Abstract

Sequential change detection is a fundamental problem in statistics and signal processing, with the CUSUM procedure widely used to achieve minimax detection delay under a prescribed false alarm rate when pre- and post-change distributions are fully known. However, in many practical settings, raw observations cannot be shared with a trusted central curator, and privacy must be enforced at the data source, which prevents the computation of exact CUSUM statistics. We therefore introduce a local differentially private (DP) variant called LDP-CUSUM, which first applies a local DP mechanism to transform the raw data into privatized observations and then applies a CUSUM procedure to detect the change. We derive closed-form bounds on the average run length to false alarm and on the worst-case average detection delay, explicitly characterizing the tradeoff among privacy level, false alarm rate, and detection efficiency. Numerical simulations and a real-data case study were conducted to demonstrate the detection efficiency of our proposed LDP-CUSUM across various scenarios.

## 1. Introduction

Sequential change detection is a fundamental problem in statistics, information theory, and signal processing, with applications across a wide range of practical domains. The canonical formulation considers a sequence of observations sampled independently, with an unknown change-point at which the underlying distribution switches from one to another. The goal of sequential change detection is to detect the occurrence of distributional change with minimal delay while controlling the false alarm rate. This problem is of major importance in many applications, such as seismic event detection [1], quality control [2], dynamical systems [3], healthcare [4], epidemiology [5], social networks [6], anomaly detection [7], detection of attacks [8], etc.

However, in many of these applications, the data streams may contain sensitive personal information, such as financial transactions or medical records [9]. In such settings, raw observations cannot be transmitted, stored, or released directly to a central monitoring system. Instead, each data point must first be privatized through a randomized mechanism before being used for inference [10,11]. Such privacy protection has also attracted substantial attention in edge-computing and task-offloading systems, where sensitive information such as user location and usage patterns may be exposed during online operation [12]. Existing works in this area have mainly focused on system-level privacy–latency joint optimization [13] and privacy-preserving task-offloading design [14], rather than on sequential change-point detection with statistical guarantees on false alarms and detection delay.

Traditional sequential detection procedures, which operate directly on the raw observations or their likelihood ratios [15,16,17], are therefore no longer applicable under such settings. This gives rise to several fundamental research questions. First, how should one design an appropriate privatization mechanism that satisfies a prescribed privacy constraint—such as local differential privacy—while preserving as much information about the distributional change as possible? Second, given only the privatized data, how should one construct a sequential detection procedure that remains statistically efficient and computationally tractable? The challenge lies in balancing three competing aspects: privacy protection, detection performance, and computational tractability. In this work, we aim to address this challenge by proposing a simple, locally private mechanism that admits a closed-form distribution of the privatized data, enabling tractable implementation of detection procedures and allowing for the explicit characterization of the resulting detection performance.

In this article, we adopt the notion of local differential privacy (LDP) to quantify the privacy level of the privatized observations [18,19]. Unlike the central DP model introduced by Dwork et al. [20], in which a trusted curator collects raw data and randomizes the aggregated output, LDP requires that each individual data point be randomized before entering the inference algorithm so that privacy protection is enforced at the data source. Informally, an α-LDP mechanism ensures that, for any two possible input values, the corresponding output distributions are close in a multiplicative sense controlled by α, meaning that an observer cannot reliably distinguish which input generated the released output. This decentralized privacy model has been widely studied in statistics and machine learning [19,21,22,23]. We study the problem of sequential change detection under local differential privacy constraints when both pre-change and post-change distributions are known. We propose a simple yet effective privatization mechanism that yields a closed-form marginal distribution for the privatized observations, enabling tractable construction of CUSUM-type detection procedures. We further characterize the detection performance under privacy constraints and quantify the tradeoff between privacy level and detection delay.

Our main contributions can be summarized as follows:

- We study sequential change detection under local differential privacy constraints and propose a simple privatization mechanism based on a bounded transformation and the Laplace mechanism. The resulting privatized observations admit a closed-form marginal distribution, which enables explicit calculations of detection statistics.
- Leveraging the closed-form structure, we construct a CUSUM-type detection statistic that operates directly on the privatized observations. The procedure is computationally efficient and suitable for online implementation. More specifically, both the privatization step and the recursive update of the detection statistic require only a constant number of operations per new sample, so the online time complexity is O(1) per step and the memory complexity is also O(1).
- We provide explicit bounds on the average run length and the detection delay of the resulting detection procedures. Our results quantify how the privacy level α impacts detection performance and show that the proposed method achieves the optimal rate up to constant factors.
- Simulation studies in various settings and a real-data case study demonstrate the effectiveness of the proposed approach and illustrate its performance relative to natural baseline privatization schemes.

The remainder of this paper is organized as follows: Section 2 provides preliminaries about sequential change detection when both pre-change and post-change distributions are fully specified and introduces the concept of α-Local DP privacy mechanism. Section 3 presents a general CUSUM-type detection procedure on privatized data and proposes a simple yet effective privacy mechanism and the induced LDP-CUSUM detection procedure. We also provide the theoretical analysis in terms of the average run length and detection delay. Section 4 presents simulation examples under a variety of univariate and multivariate distributions and a real-data case study to demonstrate the performance of the proposed LDP-CUSUM procedure. Finally, Section 5 concludes the paper and discusses directions for future work.

### Related Work

The study of sequential change detection can be traced back to the early work of Page [15] and has been studied extensively for several decades. Most works address the detection problem under the assumption of independent observations, particularly when new methods and theories are first introduced, but significant progress has also been made in extending these methods and theories to more complicated data models; see [24,25,26,27,28,29,30] for thorough reviews in this field.

The sequential detection literature can be divided into the Bayesian and the non-Bayesian (minimax) settings. The first optimality result for sequential change detection appears in [31] under a Bayesian framework, where the change-point was modeled as an independent, exponentially distributed random variable. In contrast, the non-Bayesian (minimax) setting considers the change-point to be deterministic but unknown. Under the minimax setting, the CUSUM procedure is the most widely adopted detection algorithm for the classical setup of detecting a change from a known distribution to a known alternative [15]. The CUSUM procedure was first proved to be asymptotically optimum in [32] when observations are i.i.d. before and after the change. Its exact optimality under the same data model was established in [33]. In addition to its strong optimality guarantees, the CUSUM statistic admits a recursive update, making it computationally efficient for sequential settings that require processing each new sample immediately. Although the problem of change detection when both pre-change and post-change distributions are known is fully studied in the literature, these procedures utilize raw data for constructing detection statistics and thus do not offer any privacy-preserving guarantees.

Differential privacy (DP), introduced by Dwork et al. [20], provides a rigorous framework for protecting individual data in statistical analysis. Under the central DP model, a trusted curator collects raw data and releases privatized outputs. A large body of work has adapted classical statistical procedures to satisfy privacy guarantees, including estimation and hypothesis testing [9,34,35,36,37,38,39]. In the context of sequential change detection, Cummings et al. [40] proposed an ϵ-DP procedure for estimating the change-point when both pre- and post-change distributions are specified. Their method applies a sliding-window approach combined with an offline private estimator. Extensions to settings with unknown post-change distributions were further studied in [41]. A recent work [42] studied sequential change detection under an ϵ-differential privacy constraint for single-stream data and was among the first to characterize the impact of the privacy parameter ϵ on key performance metrics such as the average run length and the worst-case average detection delay. However, all of these works operate under the central DP model, in which raw observations are shared directly with a central monitoring system.

In contrast, the local differential privacy (LDP) model [18] requires each observation to be privatized before being used for inference, eliminating the need for a trusted curator. LDP has been extensively studied in estimation and learning [19,21,43] and is particularly relevant in decentralized or streaming environments [44]. For sequential inference under LDP, Berrett and Yu [45] studied change detection in multivariate nonparametric regression models with locally privatized responses. Beyond classical change detection, privacy-preserving dynamic network analysis has been explored in [46], highlighting the additional complexity introduced by temporal dependence under privacy constraints. More recently, Asoodeh et al. [47] established an equivalent characterization of local differential privacy in terms of contraction of *f*-divergences and derived general lower bounds for estimation and hypothesis testing under LDP constraints using information-theoretic tools. In a recent work, Yadav et al. [48] studied parametric change-point detection in the offline setting under local differential privacy. They propose a locally differentially private algorithm based on randomized response and binary mechanisms and analyze their theoretical performance in terms of offline change-point estimation accuracy.

Despite these advances, theoretical characterizations of quickest detection performance under LDP—specifically in terms of average run length and worst-case detection delay—remain limited. Our work contributes to this direction by proposing a tractable locally private mechanism that enables explicit likelihood-ratio construction and by providing theoretical performance guarantees for the resulting CUSUM-type procedure.

## 2. Preliminaries and Problem Setup

In this section, we provide the necessary preliminaries and background on the sequential change detection problem and differentially private tools, then introduce the problem setup for locally differentially private sequential detection.

### 2.1. Basics of Classical Sequential Change Detection

Suppose that we observe data stream $\left\{X_{t},t\in N\right\}$, where each $X_{t}\in R^{k}.$ Initially, observations are independently and identically distributed (i.i.d.) following the probability density function (pdf) $f_{0}.$ At some unknown time τ, an event occurs and changes the distribution of the data after the time to a distinct pdf $f_{1}.$ That is,

(1) $$X_{t}\overset{i.i.d.}{∼}\left\{\begin{array}{cc} f_{0}, & t=1,2,\ldots ,\tau ,\\ f_{1}, & t=\tau +1,\tau +2,\ldots \end{array}\right.$$

In this article, we assume that $f_{0}$ and $f_{1}$ are both *known*. Here, τ∈{0,1,2,…} is a deterministic but unknown change time, and the goal is to raise the alarm as quickly as possible after the change has occurred while properly controlling the false alarm rate [25,27,29].

A sequential change detection procedure consists of a *stopping time T*, which denotes the time we stop and declare that a change has occurred before time *T*. Here, *T* is an integer-valued random variable, and the decision {T=t} is based only on the observations in the first *t* time steps. That is, $\left\{T=t\right\}\subseteq F_{t}$, where we define the filtration $F_{t}=\sigma \left\{X_{1},\ldots ,X_{t}\right\}$ and let $F_{0}$ denote the trivial sigma-algebra. To evaluate the performance of T, we further denote by $P_{\infty }$ and $E_{\infty }$ the probability measure and corresponding expectation when all samples follow the pre-change distribution $f_{0}$ (i.e., the change occurs at *∞*). Similarly, we use $P_{0}$ and $E_{0}$ to represent the probability measure and expectation when all samples follow the post-change distribution $f_{1}$ (i.e., the change occurs at 0). More generally, we denote by $P_{\tau }$ and $E_{\tau }$ the probability measure and expectation when the change happens at time τ. Under the classical minimax formulation for the sequential change detection problem [32,33], the optimal detection procedure is the one solving the following constrained optimization problem:

(2) $$\begin{array}{cc} & \underset{T}{\inf}\;\mathrm{WADD}\left(T\right):=\underset{\tau \geq 0}{\sup}\;\mathrm{ess sup}\;E_{\tau }\left[{\left(T-\tau \right)}^{+}|F_{\tau }\right]\\ & \;\mathrm{subject}\;\mathrm{to}:\;E_{\infty }\left[T\right]\geq \gamma >1. \end{array}$$

That is, among all stopping times that have an average false alarm period (also known as *average run length*, ARL) no smaller than a pre-specified constant γ>1, the optimal procedure should have the smallest *worst-case average detection delay* (WADD). Here, we adopt Lorden’s definition of WADD, which takes the supremum, over all possible change-points τ of the expected detection delay conditioned on the worst possible realizations before the change.

In the literature, it has been shown that the classical CUSUM procedure can solve the optimization problem in Equation (2) [32,33]. To be more concrete, the CUSUM statistic $\left\{S_{t},t\geq 1\right\}$ corresponds to the maximum log-likelihood ratio over all possible change-points up to time *t* and can be calculated by a recursive form:

(3) $$\begin{array}{c} S_{t}=\max_{1\leq k\leq t}\sum_{j=k}^{t}\ell \left(X_{j}\right)=\max\left(0,S_{t-1}\right)+\ell \left(X_{t}\right),\;S_{0}=0. \end{array}$$

where $\ell \left(X\right)=\log(f_{1}\left(X\right)/f_{0}\left(X\right))$ is the log-likelihood ratio (LLR) function between $f_{1}$ and $f_{0}$. The CUSUM procedure is then defined as the first time when the CUSUM statistic exceeds some pre-defined threshold *b*. That is, the CUSUM procedure is given by

(4) $$T\left(b\right)=\inf\left\{t>0:S_{t}\geq b\right\},$$

where *b* is a detection threshold specified by controlling the false alarm rate. For completeness, we provide in the following Lemma an asymptotic expression for the performance of the CUSUM procedure, which also serves as the information-theoretic lower bound for the WADD in problem (2). The proof of the following Lemma can be found in [49], Lemma 1.

**Lemma** **1** (Performance of exact CUSUM [49])**.** *For threshold $b=b_{\gamma }=\log\gamma$, the CUSUM procedure in Equation* (4) *satisfies*

(5) $$E_{\infty }\left[T\left(b_{\gamma }\right)\right]\geq \gamma ,\;\;\;\;E_{0}\left[T\left(b_{\gamma }\right)\right]=\frac{\log\gamma }{I_{0}}\left(1+o\left(1\right)\right),$$

*where $I_{0}=KL\left(f_{1}∥f_{0}\right)=E_{0}\left[\ell (X)\right]$ is the Kullback-Leibler information number (divergence) of the post- and pre-change distributions.*

It is worthwhile to note that the detection delay characterized by $E_{0}\left[T\left(b_{\gamma }\right)\right]$ in Equation (5) is the worst-case average detection delay defined in the minimax problem (2), since it is well known that the CUSUM procedure attains its worst-case delay when τ=0 [32,33]. From Lemma 1, we conclude that by applying the CUSUM procedure defined in Equation (4) with threshold b=logγ, the corresponding CUSUM stopping time *T* enjoys an asymptotic performance captured by Equation (5). By the optimality of the CUSUM procedure [33], no other stopping time $T^{\prime }$ that satisfies the same false alarm constraint can have a limiting value for the ratio $E_{0}\left[T^{\prime }\right]/\frac{\log\gamma }{I_{0}}$ that is smaller than 1 as γ→∞.

### 2.2. Local Differential Privacy

Classical sequential change detection procedures, including the CUSUM method, rely on direct access to raw observations to compute detection statistics. In many modern data collection systems, however, such centralized access is infeasible due to privacy constraints, as individual users may be unwilling or legally unable to share their unprotected data with a central curator. Instead, each participant may transmit only a randomized or sanitized version of their observation, typically under the framework of local differential privacy (LDP) [18]. Consequently, detection procedures designed for raw data are generally no longer applicable, and new methodologies must explicitly account for the privacy mechanism embedded in the data stream.

Let us first review the definition of local DP (LDP) in the literature [18]. Let X be the data domain (e.g., $X=R^{k}$), and let a database $D=\left\{X_{1},\ldots ,X_{n}\right\}\in X^{n}$ consist of *n* entries drawn from X. Under the local privacy setting, a privacy mechanism independently maps each raw data point $X_{i}\in X$ to a privatized value $Z_{i}\in Z$, where Z denotes the output (privatized) domain. Consequently, the privacy mechanism transforms the raw database $\left\{X_{1},\ldots ,X_{n}\right\}\in X^{n}$ into a privatized dataset $\left\{Z_{1},\ldots ,Z_{n}\right\}\in Z^{n}$. We consider the case when the privacy mechanism is sequentially non-interactive, meaning that each $Z_{i}$ depends only on $X_{i}$. In that case, the privacy mechanism can be specified in terms of the conditional distributions $Q(Z_{i}|X_{i}=x_{i}).$ Then, local differential privacy involves placing some restrictions on the conditional distribution *Q*, as defined in the following:

**Definition** **1** (α-LDP)**.** *For a given privacy parameter α≥0, the random variable $Z_{i}$ is an α-differentially locally private view of $X_{i}$ if for any subset of S⊆Z,*

(6) $$\begin{array}{c} \underset{x,x^{\prime }\in X}{\sup}\frac{Q(Z_{i}\in S|X_{i}=x)}{Q(Z_{i}\in S|X_{i}=x^{\prime })}\leq e^{\alpha }. \end{array}$$

Here, a larger value of α implies a weaker privacy constraint. Intuitively, the above definition means that the privacy mechanism, characterized by the distribution *Q*, perturbs the raw data in a way such that one cannot easily infer the raw data $X_{i}$ from the privatized value $Z_{i}$. Our goal is to devise a sequential detection procedure that operates on the privatized data streams $\left\{Z_{t},t\in N\right\}$ while maintaining good detection performance with a small detection delay when the average run length is controlled.

## 3. General CUSUM-Type Procedure

In this section, we begin by formalizing a CUSUM-type sequential detection procedure that operates on privatized observations, assuming that the privacy mechanism *Q* is known. In contrast to the classical setting, where likelihood ratios are computed directly from raw data, the detector here relies on likelihood ratios derived from privatized data. We then propose a simple yet general dimension-free privatization mechanism that satisfies α-local differential privacy (α-LDP). Under this mechanism, we analyze the theoretical properties of the resulting procedure and establish performance guarantees that characterize the tradeoff between privacy and detection efficiency.

### 3.1. CUSUM-Type Procedure Under General Privacy Mechanisms

In this article, we consider a special case of non-interactive local differential privacy mechanism in which each raw observation $X_{t}\in X$ is passed through the same local randomization channel *Q*, which satisfies the α-LDP constraint in Equation (6) and the corresponding randomized private sample $Z_{i}$ depends only on $X_{i}.$ Let q(z∣X=x) denote the conditional density (or probability mass function) of the privatized output *Z* given X=x. Under this mechanism, the detector does not observe $X_{t}$ directly, but instead receives the privatized independent data sequence $\left\{Z_{t},t\in N\right\}$.

Recall that $f_{0}$ and $f_{1}$ denote the pre- and post-change distributions of the raw data, respectively. The corresponding marginal distributions of the privatized observation are then given by

(7) $$m_{i}\left(z\right)=\int_{X}q\left(z|x\right)f_{i}\left(x\right)dx,\;i=0,1.$$

That is, the distribution of the privatized independent data stream $\left\{Z_{t},t\in N\right\}$ changes from $m_{0}$ to $m_{1}$ after the change-point, and $m_{0}$, $m_{1}$ serve as the effective pre- and post-change distributions available to the detector.

Based on the privatized observations, we define the corresponding CUSUM statistic ${\left\{{\tilde{S}}_{t}\right\}}_{t\geq 1}$ recursively as

(8) $$\begin{array}{c} {\tilde{S}}_{t}=\max\left(0,{\tilde{S}}_{t-1}\right)+\log\left(\frac{m_{1}\left(Z_{t}\right)}{m_{0}\left(Z_{t}\right)}\right),\;{\tilde{S}}_{0}=0. \end{array}$$

The detection procedure is defined as

(9) $$\begin{array}{cc} \tilde{T}\left(b\right) & =\inf\{t>0:{\tilde{S}}_{t}\geq b\}, \end{array}$$

where *b* is a pre-specified deterministic threshold chosen to satisfy the false alarm rate constraint.

By existing theoretical properties of the CUSUM procedure, it is easy to show the following guarantee for the CUSUM procedure $\tilde{T}\left(b\right)$ on privatized data. The proof of the following Lemma is similar to the proof of Lemma 1 in [49].

**Lemma** **2** (Performance of CUSUM Procedure $\tilde{T}\left(b\right)$)**.** *For threshold $b=b_{\gamma }=\log\gamma$, the CUSUM-type procedure in Equation* (9)*, constructed from the privatized observations, satisfies*

(10) $$E_{\infty }\left[\tilde{T}\left(b_{\gamma }\right)\right]\geq \gamma ,\;\;\;\;E_{0}\left[\tilde{T}\left(b_{\gamma }\right)\right]=\frac{\log\gamma }{{\tilde{I}}_{0}}\left(1+o\left(1\right)\right),$$

*where ${\tilde{I}}_{0}=KL\left(m_{1}∥m_{0}\right)=\int_{Z}\log\left(m_{1}\left(z\right)/m_{0}\left(z\right)\right)m_{1}\left(z\right)dz$ is the Kullback-Leibler information number (divergence) of the post- and pre-change distributions of the privatized stream.*

By the optimality of the CUSUM procedure [33], we conclude that, for a given privacy mechanism *Q* and the induced distributions $m_{1},m_{0}$, the CUSUM-type procedure in Equation (9) is the optimal detection procedure. That is, no other stopping time $T^{\prime }$ constructed on the privatized stream $\left\{Z_{t},t\in N\right\}$ which satisfies the same false alarm constraint can have a limiting value for the ratio $E_{0}\left[T^{\prime }\right]/\frac{\log\gamma }{{\tilde{I}}_{0}}$ that is smaller than 1 as γ→∞. Additionally, by the data processing inequality, we have ${\tilde{I}}_{0}=KL\left(m_{1}∥m_{0}\right)\leq KL\left(f_{1}∥f_{0}\right)=I_{0}$. That is, the KL divergence between the privatized distributions cannot exceed the KL divergence between the original distributions. Consequently, the optimum detection performance under privatization is necessarily degraded compared to the non-private setting, since the effective information number governing the detection delay is reduced.

Furthermore, Lemma 2 shows that the optimal detection performance based on the privatized data is governed by the KL divergence between the induced privatized distributions $m_{1}$ and $m_{0}$. Therefore, to achieve the best detection performance under privacy constraints, one should design the privacy mechanism—namely, the conditional distribution *Q*—so that it satisfies the α-LDP requirement while maximizing the resulting KL divergence between $m_{1}$ and $m_{0}$ after privacy transformation. To understand the fundamental limits, we first present the following lemma, which gives an upper bound for the KL divergence $KL(m_{1}∥m_{0})$ for any privacy mechanism that satisfies α-LDP.

**Lemma** **3** (Upper Bound for ${\tilde{I}}_{0}$)**.** *For the privatized data distribution $m_{0}$ and $m_{1}$ as in Equation* (7)*, we have*

$$KL\left(m_{1}∥m_{0}\right)\leq \min\left\{c_{\alpha }{\left(e^{\alpha }-1\right)}^{2}TV{\left(f_{0},f_{1}\right)}^{2},\;KL\left(f_{1}∥f_{0}\right)\right\},$$

*where $c_{\alpha }=\min\left(4,e^{2\alpha }\right)$ and $TV\left(f_{0},f_{1}\right):=\frac{1}{2}\int_{X}\left|f_{1}\left(x\right)-f_{0}\left(x\right)\right|\;dx$ is the Total-Variation divergence between the original data distributions $f_{0}$ and $f_{1}$.*

**Proof of Lemma** **3.**  The proof follows from [19], Theorem 1, which establishes a contraction inequality for α-locally differentially private channels. Let *Q* be an α-LDP mechanism and let $m_{i}$ denote the marginal distribution of the privatized observation *Z* when the raw data *X* follows distribution $f_{i}$, i=0,1. By [19], Theorem 1, for any pair of input distributions $f_{0}$ and $f_{1}$, the induced marginals satisfy

$$KL\left(m_{1}∥m_{0}\right)+KL\left(m_{0}∥m_{1}\right)\;\leq \;\min\left\{4,e^{2\alpha }\right\}{\left(e^{\alpha }-1\right)}^{2}\;TV{\left(f_{0},f_{1}\right)}^{2}.$$

Since $KL\left(m_{1}∥m_{0}\right)\leq KL\left(m_{1}∥m_{0}\right)+KL\left(m_{0}∥m_{1}\right)$, and $KL\left(m_{1}∥m_{0}\right)\leq KL\left(f_{1}∥f_{0}\right)$ by the data processing inequality, we obtain the results in Lemma 3.    □

Lemma 3 implies that, for any privacy mechanism satisfying α-LDP, the induced KL divergence between $m_{1}$ and $m_{0}$ is upper bounded by $c_{\alpha }{\left(e^{\alpha }-1\right)}^{2}TV{\left(f_{0},f_{1}\right)}^{2}$. Consequently, the optimal detection delay of any subsequent procedure based on the privatized data $Z_{t}$ is lower bounded by $\frac{\log\gamma }{c_{\alpha }{\left(e^{\alpha }-1\right)}^{2}TV{\left(f_{0},f_{1}\right)}^{2}}\left(1+o\left(1\right)\right)$. In the following subsection, we propose a specific privacy mechanism that satisfies α-LDP, is simple to implement, and achieves a detection delay matching this lower bound up to a constant factor asymptotically as α→0.

### 3.2. Proposed Privacy Mechanism and Theoretical Guarantees

We propose to construct a locally differentially private mechanism by combining a likelihood-ratio-based indicator with the Laplace mechanism. Specifically, we first reduce the raw observation $X_{t}$ to a binary statistic that indicates the most likely distribution given this single observation. That is, define the region

(11) $$A=\{x:f_{1}\left(x\right)>f_{0}\left(x\right)\},$$

which corresponds to the Neyman–Pearson most powerful test between $f_{0}$ and $f_{1}$. We then privatize the indicator of this region via additive Laplace noise. That is, the released private data $Z_{t}$ is constructed as

(12) $$\begin{array}{cc} Z_{t} & =I\left(X_{t}\in A\right)+\mathrm{Lap}\left(\frac{1}{\alpha }\right),\;\forall t\in N, \end{array}$$

where I(·) denotes the indicator function and Lap(1/α) is a Laplace random variable with mean zero and scale parameter 1/α. Since the indicator has global sensitivity equal to 1, this mechanism satisfies α-LDP by the standard Laplace mechanism. It is worth noting that such a privacy mechanism is simple to implement and applicable to data distributions of any dimension.

Furthermore, under the pre- and post-change distributions $f_{0}$ and $f_{1}$, respectively, the induced density of $Z_{t}$ can be explicitly characterized. Let

(13) $$p_{i}=P_{X∼f_{i}}\left(X\in A\right),\;i=0,1$$

denote the probability of the informative region A under distributions $f_{0}$ and $f_{1}$. Then the marginal density of *Z* under $f_{i}$ is given by the mixture

(14) $$\begin{array}{cc} m_{i}\left(z\right) & =\frac{\alpha }{2}\left[\left(1-p_{i}\right)e^{-\alpha |z|}+p_{i}e^{-\alpha |z-1|}\right],\;i=0,1. \end{array}$$

Thus, the privatized distribution is a two-component Laplace mixture, where the mixture weights $p_{i}$ encode the discrepancy between $f_{0}$ and $f_{1}$ through the likelihood-ratio region A. This explicit form enables the tractable calculation of the resulting CUSUM detection statistics and the theoretical analysis of the detection delay under the proposed locally private mechanism.

Therefore, the corresponding CUSUM-type detection statistics can be defined recursively by

(15) $$\begin{array}{c} {\tilde{S}}_{t}=\max\left(0,{\tilde{S}}_{t-1}\right)+\log\left(\frac{\left(1-p_{1}\right)e^{-\alpha |Z_{t}|}+p_{1}e^{-\alpha |Z_{t}-1|}}{\left(1-p_{0}\right)e^{-\alpha |Z_{t}|}+p_{0}e^{-\alpha |Z_{t}-1|}}\right),\;\;{\tilde{S}}_{0}=0. \end{array}$$

The detection procedure is then performed as in Equation (9) with threshold *b* chosen to satisfy the false alarm rate constraint. We summarize the overall algorithm under such a privacy mechanism in Algorithm 1, which we call the LDP-CUSUM procedure. Since the statistic is updated recursively, each new sample only requires evaluating the privatization rule and performing a constant number of arithmetic operations. Therefore, the online implementation has O(1) time complexity per step and O(1) memory complexity.
**Algorithm 1** LDP-CUSUM Procedure**Input:** Data sequence $\left\{X_{t},t\in N\right\}$, privacy parameter α, threshold b, the set A defined in (11), and $p_{0},p_{1}$ as in (13).**Output:** Stopping time $\tilde{T}\left(b\right)$.
1:Initialize t←0, ${\tilde{S}}_{0}\leftarrow -\infty$.2:**while** ${\tilde{S}}_{t}<b$ **do**3:   t←t+1 and observe a new data $X_{t}$.4:   Privatize the raw data $Z_{t}=I\left(X_{t}\in A\right)+\mathrm{Lap}\left(\frac{1}{\alpha }\right)$.5:   Update CUSUM statistic ${\tilde{S}}_{t}=\max\left(0,{\tilde{S}}_{t-1}\right)+\log\left(\frac{\left(1-p_{1}\right)e^{-\alpha |Z_{t}|}+p_{1}e^{-\alpha |Z_{t}-1|}}{\left(1-p_{0}\right)e^{-\alpha |Z_{t}|}+p_{0}e^{-\alpha |Z_{t}-1|}}\right)$.6:**end while**7:Output stopping time $\tilde{T}\left(b\right)=t$; declare that a change has occurred before time $\tilde{T}\left(b\right)$.

To analyze the theoretical performance of this detection procedure, we first establish a closed-form expression for the total variation distance between the privatized distributions $m_{1}$ and $m_{0}$ in the following Lemma:

**Lemma** **4** (TV Distance)**.** *The Total Variation (TV) distance between $m_{0}$ and $m_{1}$, under the privacy mechanism in Equation* (12)*, is given by*

(16) $$\begin{array}{c} TV\left(m_{0},m_{1}\right)=\left|p_{0}-p_{1}\right|\left(1-\exp(-\alpha /2)\right)=TV\left(f_{0},f_{1}\right)\left(1-\exp(-\alpha /2)\right). \end{array}$$

**Proof of Lemma** **4.**  From the mixture representation of $m_{0},m_{1}$ in Equation (14), we have

$$\begin{array}{cc} TV(m_{0},m_{1}) & =\frac{1}{2}\int_{R}\left|m_{1}\left(z\right)-m_{0}\left(z\right)\right|\;dz\\ & =\frac{1}{2}\cdot \frac{\alpha }{2}\cdot \int_{R}\left|\left[\left(p_{0}-p_{1}\right)e^{-\alpha |z|}+\left(p_{1}-p_{0}\right)e^{-\alpha |z-1|}\right]\right|\;dz\\ & =\frac{\alpha }{4}\left|p_{1}-p_{0}\right|\int_{R}|e^{-\alpha |z-1|}-e^{-\alpha |z|}|\;dz. \end{array}$$

Therefore, it remains to evaluate the integral $\int_{R}|e^{-\alpha |z-1|}-e^{-\alpha |z|}|\;dz$. Observe that $e^{-\alpha |z|}$ and $e^{-\alpha |z-1|}$ are symmetric Laplace kernels centered at 0 and 1, respectively. The two functions are equal at z=1/2 by symmetry. Hence, the absolute difference can be integrated by splitting at z=1/2:

$$\int_{R}|e^{-\alpha |z-1|}-e^{-\alpha |z|}|\;dz=2\int_{-\infty }^{1/2}\left(e^{-\alpha |z|}-e^{-\alpha |z-1|}\right)\;dz=\frac{4}{\alpha }\left(1-e^{-\alpha /2}\right).$$

Substituting it back yields the first equality in Equation (16). The second equality follows from the identity $|p_{1}-p_{0}|=TV\left(f_{0},f_{1}\right)$, since A is the Neyman–Pearson most powerful region. This completes the proof.    □

Building on the above lemma, we now present the main theoretical guarantees for the proposed LDP-CUSUM procedure in the following theorem:

**Theorem 1**  (Performance Guarantees)**.** *For threshold $b=b_{\gamma }=\log\gamma$, the CUSUM-type procedure in Algorithm 1 satisfies*

(17) $$E_{\infty }\left[\tilde{T}\left(b_{\gamma }\right)\right]\geq \gamma ,\;\;\;\;E_{0}\left[\tilde{T}\left(b_{\gamma }\right)\right]\leq \frac{\log\gamma }{2TV{\left(f_{0},f_{1}\right)}^{2}{\left(1-\exp\left(-\alpha /2\right)\right)}^{2}}\left(1+o\left(1\right)\right).$$

**Proof of Theorem** **1.**  The ARL lower bound $E_{\infty }\left[\tilde{T}\left(b_{\gamma }\right)\right]\geq \gamma$ follows by Lemma 1 directly. For the detection delay, we have, by Pinsker’s Inequality,

(18) $$\begin{array}{c} KL\left(m_{1}∥m_{0}\right)\geq 2TV{\left(m_{0},m_{1}\right)}^{2}=2TV{\left(f_{0},f_{1}\right)}^{2}{\left(1-\exp(-\alpha /2)\right)}^{2}, \end{array}$$

where the last equality follows from Lemma 4. Therefore, by Lemma 2, we have

$$E_{0}\left[\tilde{T}\left(b_{\gamma }\right)\right]=\frac{\log\gamma }{KL(m_{1}∥m_{0})}\left(1+o\left(1\right)\right)\leq \frac{\log\gamma }{2TV{\left(f_{0},f_{1}\right)}^{2}{\left(1-\exp(-\alpha /2)\right)}^{2}}\left(1+o\left(1\right)\right).$$

This completes the proof.    □

Comparing the lower bound of $KL\left(m_{1}∥m_{0}\right)\geq 2TV{\left(f_{0},f_{1}\right)}^{2}{\left(1-e^{-\alpha /2}\right)}^{2}$ in Equation (18) with the general upper bound in Lemma 3 that $KL\left(m_{1}∥m_{0}\right)\leq c_{\alpha }{\left(e^{\alpha }-1\right)}^{2}TV{\left(f_{0},f_{1}\right)}^{2},$ where $c_{\alpha }=\min\left(4,e^{2\alpha }\right),$ we can observe that, as α→0, the lower bound and upper bound have the same order of α up to a constant. This indicates that the detection performance under our proposed local DP mechanisms can match the information-theoretic lower bound in the first order up to a constant as α→0.

**Remark** **1** (Examples)**.** *We use a Gaussian example to illustrate the established theoretical guarantees. We first consider the example of the univariate Gaussian mean shift. Assume the pre-change distribution $f_{0}$ is $N(\mu_{0},\sigma^{2})$ and the post-change $f_{1}$ is $N(\mu_{1},\sigma^{2})$, where $\sigma^{2}$ is known and $\mu_{1}\neq \mu_{0}$. Under such settings, the total variation distance admits the closed form $TV\left(f_{0},f_{1}\right)=2\Phi (|\mu_{1}-\mu_{0}\left|/\left(2\sigma \right)\right)-1$, where Φ(·) is the cumulative distribution function of the standard normal distribution. By Lemma 3,*

$$KL\left(m_{1}∥m_{0}\right)\leq c_{\alpha }{\left(e^{\alpha }-1\right)}^{2}TV{\left(f_{0},f_{1}\right)}^{2}\overset{\mathrm{for}\;\mathrm{small}\;\alpha }{\approx }c_{\alpha }\alpha^{2}TV{\left(f_{0},f_{1}\right)}^{2}.$$

*Under our proposed local privacy mechanism, the region $A=\{x:x>\frac{\mu_{0}+\mu_{1}}{2}\}$ and by Theorem 1, the detection delay of our proposed LDP-CUSUM procedure satisfies*

$$\begin{array}{cc} & E_{0}\left[\tilde{T}\left(b_{\gamma }\right)\right]\leq \frac{\log\gamma }{2TV{\left(f_{0},f_{1}\right)}^{2}{\left(1-\exp(-\alpha /2)\right)}^{2}}\left(1+o\left(1\right)\right)\overset{\mathrm{for}\;\mathrm{small}\;\alpha }{\approx }\frac{\log\gamma }{\frac{1}{2}TV{\left(f_{0},f_{1}\right)}^{2}\alpha^{2}}\left(1+o\left(1\right)\right). \end{array}$$

**Figure 1:**
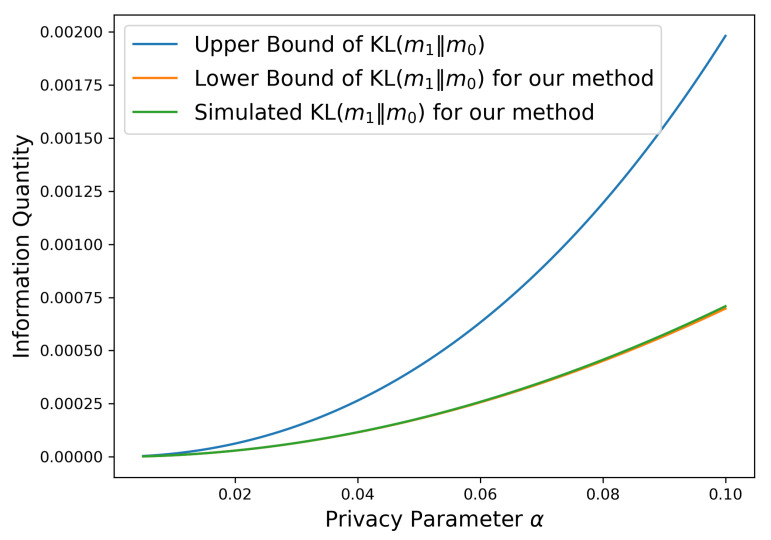
Comparison of information quantities as functions of the privacy parameter α under the univariate Gaussian mean-shift model.

*Figure 1 illustrates the behavior of the induced KL divergence as a function of the privacy parameter α under the Gaussian mean-shift model with $|\mu_{1}-\mu_{0}|=1$ and σ=1. The blue curve corresponds to the general upper bound derived in Lemma 3, which applies to any α-LDP mechanism. The orange curve shows the analytical lower bound obtained via Pinsker’s inequality for the proposed LDP-CUSUM mechanism, while the green curve represents the exact KL divergence $KL(m_{1}∥m_{0})$ computed numerically from the explicit density in Equation* (14)*. We observe that, as α→0, both the upper bound and the KL divergence of the proposed mechanism scale as $\alpha^{2}$, confirming the optimal first-order privacy–information tradeoff. Moreover, the numerically computed KL divergence, which serves as a proxy for the true $KL(m_{1}∥m_{0})$, remains within constant factors of the upper bound across the range of α, and is very close to the lower bound obtained via Pinsker’s inequality when α is small, demonstrating that the proposed mechanism is near-optimal in terms of information contraction up to constant factors. The same analysis naturally extends to the multivariate Gaussian mean-shift setting and to exponential family models in general.*

**Remark** **2** (Extensions to Other Bounded Transformations)**.** *In this work, we propose to use the indicator function in Equation* (12)* as a bounded transformation of the raw data so that the induced marginal distribution of the privatized variable $Z_{t}$ admits a closed-form expression. This explicit form enables the tractable, computationally efficient calculation of CUSUM-type statistics, thereby facilitating both implementation and theoretical analysis of the detection procedure. However, such a binary transformation may lose the information contained in the original raw data $X_{t}$. Thus, it is a future direction to design alternative bounded (and possibly smooth) transformations—such as a sigmoid-type mapping—that retain more information from X while still leading to a closed-form density, or at least a density that can be efficiently approximated, for the privatized data $Z_{t}$.*

**Remark** **3** (Comparison with Randomized Response)**.** *Randomized response (RR) is one of the most classical and widely used mechanisms for guaranteeing α-local differential privacy. For a finite alphabet X with |X|=q, the q-ary RR mechanism is defined as*

$$Q_{\mathrm{RR}}\left(z|x\right)=\left\{\begin{array}{cc} \frac{e^{\alpha }}{e^{\alpha }+q-1}, & z=x,\\ \frac{1}{e^{\alpha }+q-1}, & z\neq x. \end{array}\right.$$

*RR and its variants have been extensively used in locally private inference problems, including change-point detection under LDP [45,46,48]. In contrast, our approach is not restricted to finite-alphabet randomized response. Instead, we first allow for a general transformation T:X→[0, 1] (possibly multi-level or even continuous), followed by additive Laplace noise. This framework strictly generalizes the binary case and remains compatible with non-binary and continuous representations of the data. An important distinction arises when the alphabet size grows. For q-ary RR, as q→∞ with α fixed, the RR privacy mechanism approaches a nearly uniform mapping, and the induced KL divergence between post-change and pre-change privatized distributions can decay quickly as the alphabet enlarges. In contrast, our method can be generalized to continuous transformations that do not require operating directly on a large discrete alphabet and can preserve more distributional structure through carefully chosen transformations. Therefore, our method provides a more flexible alternative that naturally extends to non-binary and continuous transformations.*

## 4. Experimental Results

In this section, we conduct comprehensive simulation studies and a real-data case study to evaluate the performance of the proposed locally differentially private CUSUM procedure in Algorithm 1. In the simulated experiments, we compare its detection delay and false alarm behavior with baseline methods to examine how different privatization strategies affect the detection delay under a fixed false alarm constraint, and examine how the privacy level α affects statistical efficiency under different distributional settings.

### 4.1. Simulation Results Under Univariate Distributions

We first consider univariate models and compare the proposed LDP-CUSUM procedure with a natural truncation-based baseline. We first specify the natural baseline approach in the following. For a scalar observation *X*, the baseline privatization is defined by

(19) $$\begin{array}{cc} Z & ={\left[X\right]}_{-K}^{K}+\mathrm{Lap}\;\left(\frac{2K}{\alpha }\right), \end{array}$$

where ${\left[X\right]}_{-K}^{K}=\min\left\{\max\left\{X,-K\right\},K\right\}$ denotes truncation to the interval [−K,K]. Since the truncated statistic has global sensitivity 2K, adding Laplace noise with scale 2K/α guarantees α-LDP. We construct the corresponding CUSUM-type statistic as

(20) $$\begin{array}{c} {\tilde{S}}_{t}=\max\;\left(0,{\tilde{S}}_{t-1}\right)+\log\;\left(\frac{f_{1}\left(Z_{t}\right)}{f_{0}\left(Z_{t}\right)}\right),\;{\tilde{S}}_{0}=0. \end{array}$$

Note that, for this baseline method, we use the original densities $f_{0}$ and $f_{1}$ to form the log-likelihood ratio, since the exact marginal distribution $m_{i}$ of the privatized data *Z* does not admit a closed-form expression after truncation and noise addition. In addition, the truncation parameter *K* can be selected numerically in practice via simulation to optimize empirical detection performance under each configuration. In our experiments, we simulate the performance under various *K* values to demonstrate the overall performance of this truncation-type baseline approach.

It is worth noting that the truncation-based baseline is more generic at the privatization stage, since the transformation in Equation (19) can be applied without knowing the pre- and post-change distributions. However, the resulting detection rule in Equation (20) still relies on prior distributional knowledge of the pre- and post-change models. Therefore, when the post-change distribution is unknown, the current truncation-based baseline does not fully avoid this difficulty. Its main advantage is instead that the privatization mechanism itself is distribution-agnostic, whereas our indicator-based privatization rule is directly constructed from $f_{0}$ and $f_{1}$.

Our simulation is performed under three classical mean- and variance-shift models:

- Gaussian mean shift: We set the pre-change distribution as N(0,1) and the post-change distribution to be N(1,1). This yields the indicator region A=(0.5,∞), so the indicator statistic is $I(X_{t}>0.5)$. For the truncation method, we consider three truncation levels of K∈{0.8,0.9,1}.
- Laplace mean shift: We let the distribution change from Lap(0,1) to Lap(0.5,1). The likelihood-ratio equation leads to A=(0.25,∞). We again consider three truncation levels of K∈{0.8,0.9,1}.
- Gaussian variance shift: We let the distribution changes from N(0,1) to N(0,4). The corresponding decision region is A={x:|x|>1.36}. For the truncation method, we consider K∈{1.5,1.8,2.2}.

For each change model, we vary the privacy parameter α from 0.5 to 2. For each α, the detection threshold *b* of each method is calibrated to achieve an average run length of approximately ARL≈1000. The corresponding average detection delay is then simulated via Monte Carlo simulation with 1000 independent replications.

**Figure 2:**
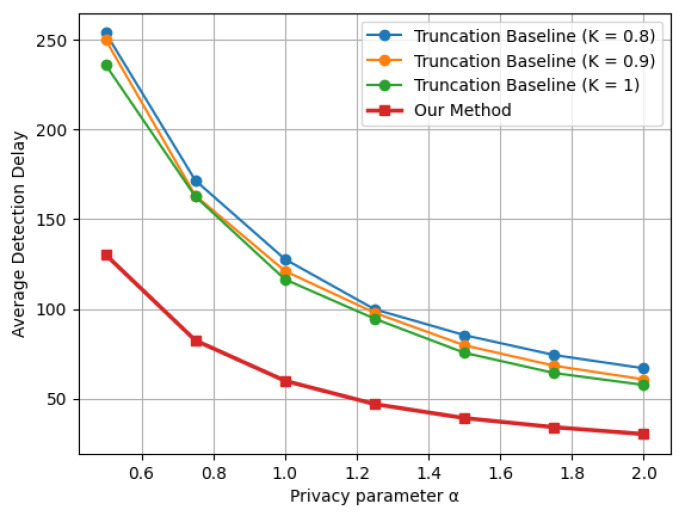
Delay–privacy tradeoff curves comparing Truncation and Indicator LDP-CUSUM under a Gaussian mean shift N(0,1)→N(1,1), with ARL≈1000.

Figure 2 presents the expected detection delay as a function of the privacy parameter α for both the proposed LDP-CUSUM procedure and the truncation-based baseline under multiple truncation levels *K*, for the Gaussian mean shift case. It can be observed that the detection delay decreases, for all methods, as α increases. This is because a larger α, by definition, implies a weaker privacy guarantee and thus less distortion of the original data distributions. This also demonstrates the fundamental tradeoff between detection efficiency and data privacy. Moreover, the proposed indicator-based LDP-CUSUM procedure consistently outperforms the truncation baseline across all considered truncation levels. The performance gap is significant across the entire range of privacy parameters, indicating that the indicator-based mechanism is more effective at preserving the signal-to-noise ratio between the pre- and post-change distributions after privatization.

**Figure 3:**
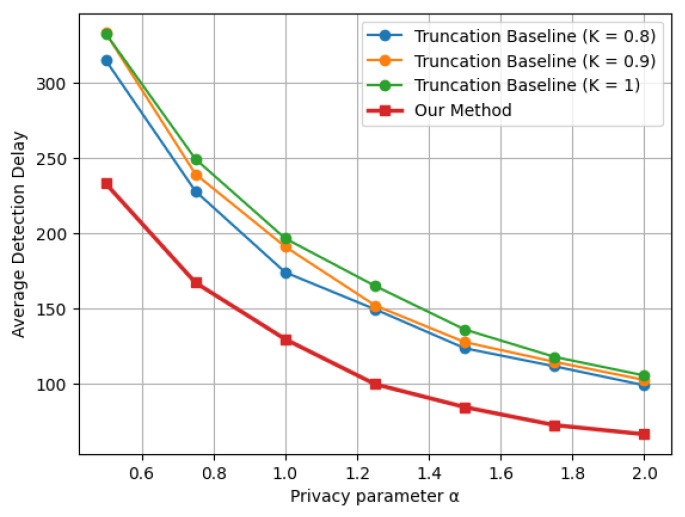
Delay–privacy tradeoff curves comparing Truncation and Indicator LDP-CUSUM under a Laplace mean shift Lap(0,1)→Lap(0.5,1), with ARL≈1000.

**Figure 4:**
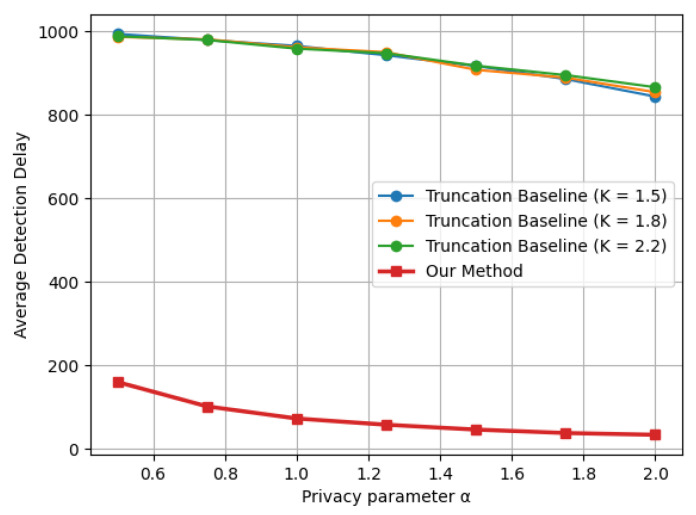
Delay–privacy tradeoff curves comparing Truncation and Indicator LDP-CUSUM under a Gaussian variance shift N(0,1)→N(0,4), with ARL≈1000.

Similar trends are observed in Figure 3 and Figure 4, corresponding to the Laplace mean shift and Gaussian variance shift settings, respectively. Across all three distributional changes considered, the proposed indicator-based LDP-CUSUM consistently achieves substantially smaller detection delay than the truncation baseline over the entire range of privacy parameters. These results confirm the robustness of the proposed mechanism across different types of structural changes. Furthermore, comparing Figure 4 with Figure 2, we observe that the truncation baseline leads to a substantially higher detection delay than the proposed method, especially for detecting variance shifts. A possible explanation is that variance shifts are primarily reflected in the tail behavior of the distribution. The truncation step thus leads to substantial information loss by clipping extreme observations, which are the most informative for detecting variance changes. In contrast, the proposed indicator-based mechanism defines the decision region based on the likelihood ratio and suffers less severe information loss, thereby maintaining stronger separation between the pre- and post-change distributions after privatization.

### 4.2. Simulation Results Under Multivariate Distributions

We then consider changes in multivariate distributions and evaluate the proposed LDP-CUSUM under Gaussian mean-shift and covariance-shift models. We examine how the proposed mechanism extends to multi-dimensional settings and compare its performance with truncation-based baselines. We first specify the truncation baseline method under the multivariate case. Let $X_{t}\in R^{k}$ be the raw observation. The truncation baseline first projects $X_{t}$ onto an $\ell_{p}$ ball of radius *K* and then adds multivariate Laplace noise [50]:

(21) $$\begin{array}{cc} Z_{t} & =\Pi_{{∥\cdot ∥}_{p}\leq K}\left(X_{t}\right)+W_{t},\;f_{W_{t}}\left(w\right)\propto \exp\;\left(-\frac{{∥w∥}_{p}}{2K/\alpha }\right), \end{array}$$

where $\Pi_{{∥\cdot ∥}_{p}\leq K}\left(\cdot \right)$ denotes the projection onto the $\ell_{p}$ ball of radius *K*. In all multivariate experiments, we use p=1, under which $W_{t}\left(j\right)\overset{i.i.d.}{∼}\mathrm{Lap}\left(2K/\alpha \right)$ for j=1,…,k. That is, we add i.i.d. Laplace noise to each dimension of the truncated data. Since the $\ell_{1}$-sensitivity of the projection is bounded by 2K, this mechanism satisfies α-LDP. We then construct the CUSUM statistic as in (20), in which we use the raw data distribution $f_{0},f_{1}$ as a surrogate since the exact marginal distribution of $Z_{t}$ does not admit a closed-form expression after projection and noise addition.

We consider the following two multivariate Gaussian models:

- Multivariate Gaussian mean shift: We set the pre-change distribution as $N(0_{k},I_{k})$ and the post-change distribution as $N(0.5\cdot 1_{k},I_{k})$, where $I_{k}$ denotes the *k*-dimensional identity matrix and $1_{k}$ denotes the *k*-dimensional vector of all ones. We consider two dimensions, k=5 and k=10, to examine the effect of increasing dimensionality on detection performance. For the proposed LDP-CUSUM procedure, we have a closed-form region $A=\{x:\;1_{k}^{⊤}x>0.25k\}$. For the truncation baseline, we compare multiple truncation radii K∈{2,4,6} to illustrate the sensitivity of detection performance to the truncation level.
- Multivariate Gaussian covariance shift: We set the pre-change distribution as $N(0_{k},I_{k})$ and the post-change distribution as $N(0_{k},\Sigma )$, with a post-change covariance matrix $\Sigma =I_{k}+ruu^{⊤},$r=0.8, and $u\in R^{k}$ is a pre-set vector. This corresponds to a rank-one spiked covariance matrix and has wide applicability in various applications such as human activity detection and community detection [51,52]. Such a distributional change also admits a closed-form expression for the region A as $A=\{x:{\left(u^{⊤}x\right)}^{2}>\frac{1+{r∥u∥}_{2}^{2}}{r}\log\;\left(1+{r∥u∥}_{2}^{2}\right)\}$. We mainly focus on k=5 when examining the impact of privacy on covariance detection.

We follow the same evaluation protocol as in Section 4.1. Specifically, for each configuration, the threshold is calibrated such that ARL≈1000 under the pre-change regime, and the corresponding average detection delay is simulated under the post-change regime.

**Figure 5:**
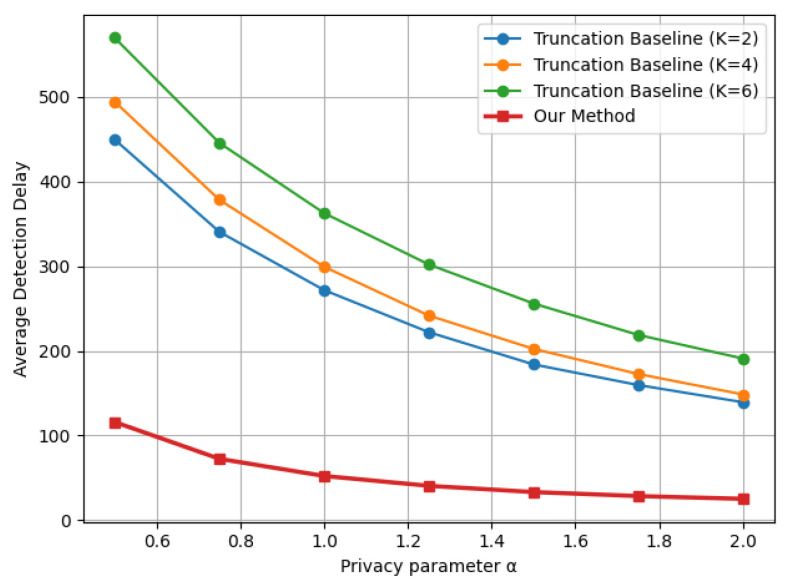
Delay–privacy tradeoff curves comparing the proposed LDP-CUSUM and the truncation-based LDP baseline under a multivariate Gaussian mean shift $N\left(0,I_{5}\right)\to N\left(0.5\cdot 1_{5},I_{5}\right)$, ARL≈1000, with the truncation baseline evaluated at K∈{2,4,6}.

**Figure 6:**
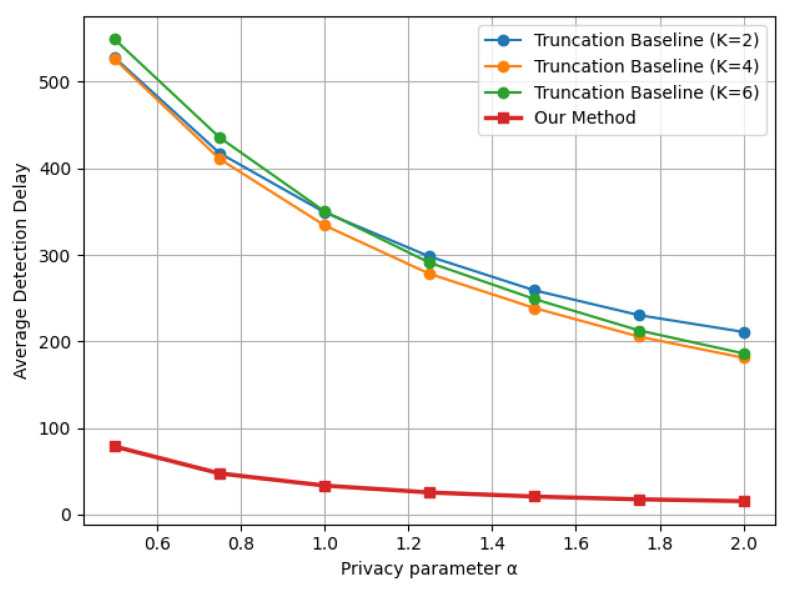
Delay–privacy tradeoff curves comparing the proposed LDP-CUSUM and the truncation-based LDP baseline under a multivariate Gaussian mean shift $N\left(0,I_{10}\right)\to N\left(0.5\cdot 1_{10},I_{10}\right)$, ARL≈1000, with the truncation baseline evaluated at K∈{2,4,6}.

Figure 5 illustrates the detection delay as a function of the privacy parameter α∈[0.5,2] for the Gaussian mean shift model with dimension k=5, while Figure 6 presents the corresponding results for k=10. In both scenarios, we observe a clear delay–privacy tradeoff that increasing α (corresponding to weaker privacy and less injected noise) monotonically reduces the detection delay for all methods. More importantly, we observe a significant performance gap across all privacy levels. For all truncation radii *K* considered, the truncation-based baseline yields noticeably larger detection delays than the proposed method. Furthermore, the truncation baseline performs similarly under different values of *K*, indicating that the performance gap remains consistent over a broad range of truncation radii and is not sensitive to the specific choice of *K*. This further highlights the advantage of the proposed method.

**Figure 7:**
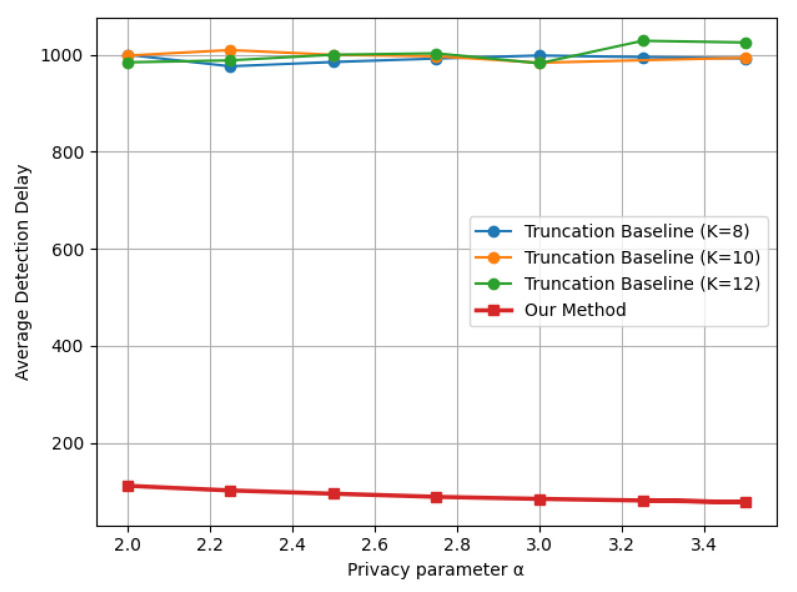
Delay–privacy tradeoff curves comparing the proposed LDP-CUSUM and the truncation-based LDP baseline under a multivariate Gaussian covariance shift $N\left(0,I_{5}\right)\to N\left(0,\Sigma \right)$, $\Sigma =I_{5}+ruu^{⊤}$, r=0.8, $u={\left[1,1.5,2,2.5,3\right]}^{⊤}$, ARL≈1000, with the truncation baseline evaluated at K∈{8,10,12}.

Figure 7 presents the results under the covariance shift model. We note that covariance changes are more challenging to detect using the truncation baseline, since projection can distort tail behavior, and the added coordinate-wise Laplace noise may mask the correlation structure; this effect is reflected in the substantially larger delay of the truncation baseline in the tradeoff plots. The proposed method mitigates these issues by employing an indicator-based privatization mechanism that better preserves change-relevant signals under LDP, thereby achieving consistently smaller detection delays.

To provide a more comprehensive view of the tradeoff between detection delay and ARL, and to further illustrate the impact of LDP constraints on detection performance, we also report delay–ARL tradeoff curves obtained by varying the detection threshold. In addition to the proposed method and the truncation-based baseline, we also compare the non-private exact CUSUM test constructed from the true log-likelihood ratio, which serves as an information-theoretical lower bound on the achievable detection delay.

**Figure 8:**
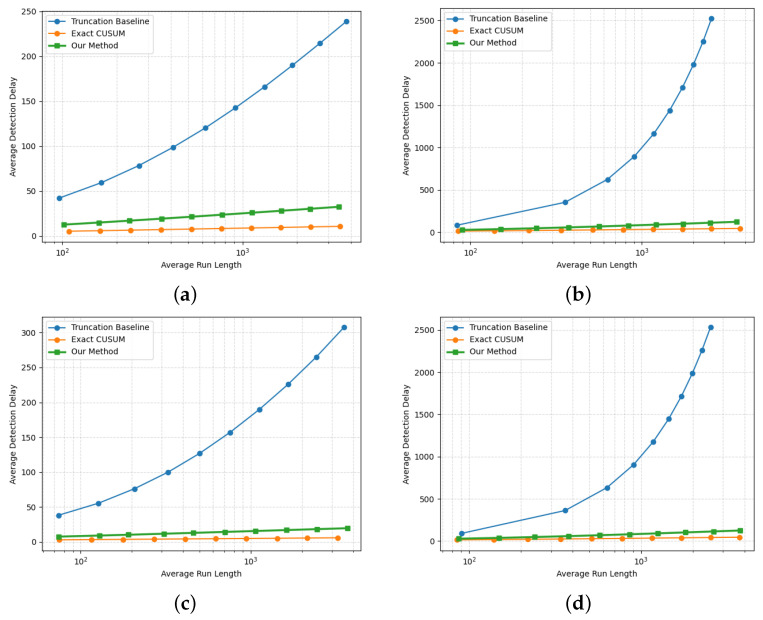
Delay–ARL tradeoff curves comparing the proposed LDP-CUSUM, the truncation-based baseline as in Equation (20), and the non-private exact CUSUM in Equation (4). (**a**) Multivariate Gaussian mean shift $N\left(0,I_{5}\right)\to N\left(0.5\cdot 1_{5},I_{5}\right)$ with α=2, K=4. (**b**) Multivariate Gaussian covariance shift $N\left(0,I_{5}\right)\to N\left(0,\Sigma \right)$, $\Sigma =I_{5}+ruu^{⊤}$, r=0.8, $u={\left[1,1.5,2,2.5,3\right]}^{⊤}$, α=3, K=10. (**c**) Multivariate Gaussian mean shift $N\left(0,I_{10}\right)\to N\left(0.5\cdot 1_{10},I_{10}\right)$, α=2, K=4. (**d**) Multivariate Gaussian covariance shift $N\left(0,I_{10}\right)\to N\left(0,\Sigma \right)$, $\Sigma =I_{10}+ruu^{⊤}$, r=0.8, $u={\left[1,1.5,2,2.5,3,3.5,4,4.5,5,5.5\right]}^{⊤}$, α=3, K=20.

Figure 8 presents the delay–ARL tradeoff curves under both mean and covariance shifts for dimensions k=5 and k=10. It can be observed that the non-private exact CUSUM achieves the smallest detection delay for any fixed ARL due to its exact optimality [33]. Among the locally private procedures, the proposed method consistently yields substantially lower detection delay than the truncation baseline across all ARL values and across all settings. Moreover, the detection delay of the proposed method is close to that of the exact CUSUM, indicating that the performance degradation induced by the LDP constraint is less severe. The gap between the proposed method and the exact CUSUM reflects the fundamental impact of local differential privacy requirements on detection efficiency. Furthermore, the results in Figure 8 indicate that covariance shifts are inherently more difficult to detect than mean shifts, both in the non-private setting and under LDP constraints.

### 4.3. A Real-Data Example

We evaluate the method on the Danmini Doorbell stream from a publicly available Internet of Things (IoT) botnet attacks (N-BaIoT) dataset [53,54]. Each observation is a time-ordered 115-dimensional numeric feature vector summarizing packet-level and flow-level traffic statistics. To obtain a compact representation for sequential detection, we apply principal component analysis to the Doorbell data to reduce the dimensionality to five, and standardize the retained components to have zero mean and unit variance. In this experiment, benign traffic is treated as the pre-change distribution, while traffic generated under the junk attack is treated as the post-change distribution. We use historical data collected under benign traffic and previous junk attacks to estimate the pre- and post-change data distributions using Gaussian models.

**Figure 9:**
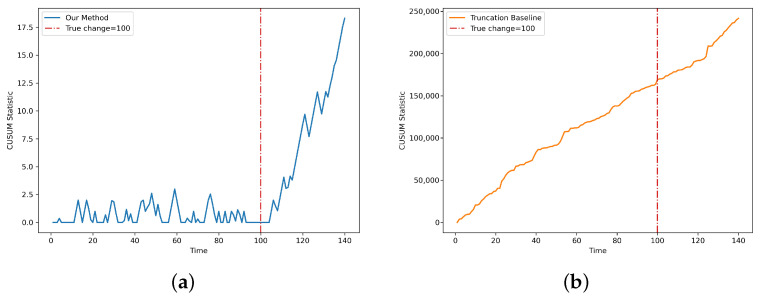
Trajectories of detection statistics for the proposed LDP-CUSUM in panel (**a**) and the truncation-based baseline in panel (**b**) on a real IoT botnet dataset with the Danmini Doorbell device during a junk attack (α=1, K=10).

Figure 9 shows the trajectories of the detection statistic for the proposed LDP-CUSUM and the truncation-based baseline on this single-device experiment under the junk attack, with privacy level α=1 and truncation parameter K=10. For our proposed method, the LDP-CUSUM statistic stays close to zero throughout the pre-change segment and then increases sharply after the true change-point, indicating that the privatized observations still preserve sufficient information for effective detection. By contrast, the truncation-based baseline shows a pronounced upward drift even before the change occurs, suggesting that its surrogate log-likelihood ratio is positively biased under the pre-change distribution. In practice, such behavior may lead to frequent false alarms. These results demonstrate that the proposed LDP-CUSUM detection procedure remains effective in practice.

## 5. Discussion and Conclusions

In this work, we studied the problem of online sequential change detection under local differential privacy constraints. Motivated by scenarios in which raw observations cannot be directly released to the central curator, we proposed a simple privatization mechanism based on a bounded transformation combined with the Laplace mechanism. The resulting privatized data admit a closed-form marginal distribution, which enables tractable implementation of CUSUM-type detection procedures.

We characterized the detection performance in terms of the induced divergence between privatized pre- and post-change distributions and quantified the tradeoff between privacy protection and detection delay. Our analysis shows that the proposed method achieves the optimal rate with respect to the privacy parameter up to constant factors asymptotically. Numerical experiments further demonstrate its effectiveness compared with natural truncation-type baseline approaches.

There are several directions for future work. First, it would be interesting to design smoother bounded transformations—for example, smooth functions that map the raw observations $X_{t}$ into [0,1] so that more information is preserved while keeping the analysis tractable. Compared with classical randomized response mechanisms, our approach has the potential to improve detection performance by choosing such smooth transformations that better retain important features (e.g., modal information) of the original data distributions. Second, a natural extension is to handle composite or unknown post-change distributions. This could be done by incorporating generalized likelihood ratio (GLR) procedures or window-limited CUSUM tests into the proposed framework. Moreover, it would also be useful to study adaptive or data-driven privatization strategies in high-dimensional settings, with the objective of reducing the impact of privacy constraints on detection delay. This could be formulated as an interactive LDP mechanism where the privatization rule at time *t* can be adapted using previously released privatized outputs. Such an interactive LDP design may improve detection performance when we have composite post-change models, temporally dependent data streams, or adaptive privacy budget allocation over time. These problems remain open for future research.

## Data Availability

The original contributions presented in this study are included in the article. Further inquiries can be directed to the corresponding author.

## References

[1] Xie L., Xie Y., Moustakides G.V. (2019). Asynchronous multi-sensor change-point detection for seismic tremors. Proceedings of the 2019 IEEE International Symposium on Information Theory (ISIT).

[2] Shi J., Zhou S. (2009). Quality control and improvement for multistage systems: A survey. IIE Trans..

[3] Lai T.L. (1995). Sequential changepoint detection in quality control and dynamical systems. J. R. Stat. Soc. Ser. B (Methodol.).

[4] Balageas D., Fritzen C.P., Güemes A. (2010). Structural Health Monitoring.

[5] Zhu S., Bukharin A., Xie L., Yamin K., Yang S., Keskinocak P., Xie Y. (2022). Early detection of COVID-19 hotspots using spatio-temporal data. IEEE J. Sel. Top. Signal Process..

[6] Li S., Xie Y., Farajtabar M., Verma A., Song L. (2017). Detecting changes in dynamic events over networks. IEEE Trans. Signal Inf. Process. Netw..

[7] Chandola V., Banerjee A., Kumar V. (2009). Anomaly detection: A survey. ACM Comput. Surv. (CSUR).

[8] Tartakovsky A.G. (2014). Rapid detection of attacks in computer networks by quickest changepoint detection methods. Data Analysis for Network Cyber-Security.

[9] Cai T.T., Wang Y., Zhang L. (2021). The cost of privacy: Optimal rates of convergence for parameter estimation with differential privacy. Ann. Stat..

[10] Seif M., Xie L., Goldsmith A.J., Poor H.V. (2025). Differentially Private Online Community Detection for Censored Block Models: Algorithms and Fundamental Limits. IEEE Trans. Inf. Forensics Secur..

[11] Teku N., Tian F., Bhattacharjee P., Chakraborty S., Bedi A.S., Tandon R. (2025). PROPS: Progressively Private Self-alignment of Large Language Models. Trans. Mach. Learn. Res..

[12] Li T., He X., Jiang S., Liu J. (2022). A survey of privacy-preserving offloading methods in mobile-edge computing. J. Netw. Comput. Appl..

[13] Zhao P., Tao J., Lui K., Zhang G., Gao F. (2022). Deep reinforcement learning-based joint optimization of delay and privacy in multiple-user MEC systems. IEEE Trans. Cloud Comput..

[14] He X., Liu J., Jin R., Dai H. (2017). Privacy-aware offloading in mobile-edge computing. Proceedings of the GLOBECOM 2017—2017 IEEE Global Communications Conference.

[15] Page E.S. (1954). Continuous Inspection Schemes. Biometrika.

[16] Lai T.L. (1998). Information Bounds and Quick Detection of Parameter Changes in Stochastic Systems. IEEE Trans. Inf. Theory.

[17] Xie L., Moustakides G.V., Xie Y. (2023). Window-limited CUSUM for sequential change detection. IEEE Trans. Inf. Theory.

[18] Kasiviswanathan S.P., Lee H.K., Nissim K., Raskhodnikova S., Smith A. (2011). What can we learn privately?. SIAM J. Comput..

[19] Duchi J.C., Jordan M.I., Wainwright M.J. (2018). Minimax optimal procedures for locally private estimation. J. Am. Stat. Assoc..

[20] Dwork C., McSherry F., Nissim K., Smith A. (2006). Calibrating noise to sensitivity in private data analysis. Proceedings of the Theory of Cryptography Conference.

[21] Kairouz P., Oh S., Viswanath P. (2014). Extremal mechanisms for local differential privacy. Adv. Neural Inf. Process. Syst..

[22] Erlingsson Ú., Pihur V., Korolova A. (2014). Rappor: Randomized aggregatable privacy-preserving ordinal response. Proceedings of the 2014 ACM SIGSAC Conference on Computer and Communications Security.

[23] Warner S.L. (1965). Randomized response: A survey technique for eliminating evasive answer bias. J. Am. Stat. Assoc..

[24] Basseville M., Nikiforov I.V. (1993). Detection of Abrupt Changes: Theory and Application.

[25] Poor H.V., Hadjiliadis O. (2008). Quickest Detection.

[26] Siegmund D. (1985). Sequential Analysis: Tests and Confidence Intervals.

[27] Tartakovsky A., Nikiforov I., Basseville M. (2014). Sequential Analysis: Hypothesis Testing and Changepoint Detection.

[28] Tartakovsky A. (2019). Sequential Change Detection and Hypothesis Testing: General Non-Iid Stochastic Models and Asymptotically Optimal Rules.

[29] Veeravalli V.V., Banerjee T. (2014). Quickest change detection. Acad. Press Libr. Signal Process. Array Stat. Signal Process..

[30] Xie L., Zou S., Xie Y., Veeravalli V.V. (2021). Sequential (quickest) change detection: Classical results and new directions. IEEE J. Sel. Areas Inf. Theory.

[31] Shiryaev A.N. (1963). On optimum methods in quickest detection problems. Theory Probab. Its Appl..

[32] Lorden G. (1971). Procedures for Reacting to a Change in Distribution. Ann. Math. Stat..

[33] Moustakides G.V. (1986). Optimal stopping times for detecting changes in distributions. Ann. Stat..

[34] Avella-Medina M. (2021). Privacy-preserving parametric inference: A case for robust statistics. J. Am. Stat. Assoc..

[35] Degue K.H., Le Ny J. (2018). On Differentially Private Gaussian Hypothesis Testing. Proceedings of the 2018 56th Annual Allerton Conference on Communication, Control, and Computing (Allerton).

[36] Canonne C.L., Kamath G., McMillan A., Smith A., Ullman J. (2019). The structure of optimal private tests for simple hypotheses. Proceedings of the 51st Annual ACM SIGACT Symposium on Theory of Computing.

[37] Zhang R. (2024). Detection of Sparse Mixtures With Differential Privacy. IEEE J. Sel. Areas Inf. Theory.

[38] Lau T.S., Tay W.P. (2020). Privacy-aware quickest change detection. Proceedings of the ICASSP 2020—2020 IEEE International Conference on Acoustics, Speech and Signal Processing (ICASSP).

[39] Lau T.S., Tay W.P. (2020). Quickest change detection with privacy constraint. arXiv.

[40] Cummings R., Krehbiel S., Mei Y., Tuo R., Zhang W. (2018). Differentially private change-point detection. Adv. Neural Inf. Process. Syst. (NeurIPS).

[41] Cummings R., Krehbiel S., Lut Y., Zhang W. Privately detecting changes in unknown distributions. Proceedings of the International Conference on Machine Learning (ICML).

[42] Xie L., Zhang R. (2026). Sequential Change Detection with Differential Privacy. IEEE Trans. Inf. Theory.

[43] Bassily R., Smith A. (2015). Local, private, efficient protocols for succinct histograms. Proceedings of the Forty-Seventh Annual ACM Symposium on Theory of Computing.

[44] Yang M., Guo T., Zhu T., Tjuawinata I., Zhao J., Lam K.Y. (2024). Local differential privacy and its applications: A comprehensive survey. Comput. Stand. Interfaces.

[45] Berrett T., Yu Y. (2021). Locally private online change point detection. Adv. Neural Inf. Process. Syst. (NeurIPS).

[46] Li M., Berrett T., Yu Y. (2022). Network change point localisation under local differential privacy. Adv. Neural Inf. Process. Syst. (NeurIPS).

[47] Asoodeh S., Aliakbarpour M., Calmon F.P. (2021). Local Differential Privacy Is Equivalent to Contraction of an *f*-Divergence. Proceedings of the 2021 IEEE International Symposium on Information Theory (ISIT).

[48] Yadav A.K., Cadir C., Shkel Y., Gastpar M. (2026). Locally Private Parametric Methods for Change-Point Detection. arXiv.

[49] Xu Q., Mei Y., Moustakides G.V. (2021). Optimum multi-stream sequential change-point detection with sampling control. IEEE Trans. Inf. Theory.

[50] Hardt M., Talwar K. (2010). On the geometry of differential privacy. Proceedings of the Forty-Second ACM Symposium on Theory of Computing.

[51] Xie L., Xie Y., Moustakides G.V. (2020). Sequential subspace change point detection. Seq. Anal..

[52] Zhang M., Xie L., Xie Y. (2023). Spectral CUSUM for Online Network Structure Change Detection. IEEE Trans. Inf. Theory.

[53] Meidan Y., Bohadana M., Mathov Y., Mirsky Y., Shabtai A., Breitenbacher D., Elovici Y. (2018). N-baiot—network-based detection of iot botnet attacks using deep autoencoders. IEEE Pervasive Comput..

[54] Kurt M.N., Yılmaz Y., Wang X., Mosterman P.J. (2022). Online privacy-preserving data-driven network anomaly detection. IEEE J. Sel. Areas Commun..

